# *survivin* messenger RNA expression is a good prognostic biomarker for oesophageal carcinoma

**DOI:** 10.1038/sj.bjc.6600546

**Published:** 2002-10-07

**Authors:** M Ikeguchi, N Kaibara

**Affiliations:** First Department of Surgery, Faculty of Medicine, Tottori University, 36-1 Nishi-cho, Yonago 683-8504, Japan

**Keywords:** oesophageal squamous cell carcinoma, *survivin*, real-time reverse transcriptase polymerase chain reaction, prognosis

## Abstract

Oesophageal squamous cell carcinoma is one of the most malignant tumours. To identify patients with a high risk of recurrence of oesophageal squamous cell carcinoma, we investigated the prognostic significance of *survivin* mRNA expression in oesophageal squamous cell carcinoma, which has recently been reported to be a good marker for unfavourable prognosis in various tumours. Tumours and non-cancerous epitheliums adjacent to tumours were obtained by surgical resection from 57 patients with oesophageal squamous cell carcinoma. Expression levels of *survivin* and *glyceraldehyde-3-phosphate dehydrogenase* mRNA were analysed quantitatively by real-time reverse transcriptase polymerase chain reaction (RT–PCR). The *survivin*/*glyceraldehyde-3-phosphate dehydrogenase* ratios of tumours were higher than those of non-cancerous tissues (*P*=0.0003). Tumour-*survivin*/*glyceraldehyde-3-phosphate dehydrogenase* ratio did not correlate with histologic type, lymph node metastasis, and stage of tumours. In 53 surviving patients, the 5-year survival rate of 17 patients with high *survivin* mRNA expressed oesophageal squamous cell carcinoma (14.1%) was significantly poorer than that of 36 with low *survivin* mRNA expressed oesophageal squamous cell carcinoma (46.8%, *P*=0.0018). In these patients, tumour-*survivin* mRNA expression was recognised as a good marker of cancer recurrence independently from tumour stage. These findings indicate that *survivin* mRNA expression in oesophageal squamous cell carcinoma may be a good biomarker for identifying patients with high risk of cancer recurrence.

*British Journal of Cancer* (2002) **87**, 883–887. doi:10.1038/sj.bjc.6600546 www.bjcancer.com

© 2002 Cancer Research UK

## 

Oesophageal squamous cell carcinoma (OSCC) is one of the most malignant tumours having a dismal prognosis. Lymph node metastasis has a strong prognostic impact for patients with OSCC (Tachibana *et al*, 1999; Hosch *et al*, 2001). However, even in early stage OSCC, many patients develop recurrent tumours that once developed indicate a poor patient prognosis. Starting adequate adjuvant chemo-radiotherapy for patients with a high risk of cancer recurrence is required. In order to identify patients with a high risk of cancer recurrence, a variety of biologic markers have been investigated (Sarbia *et al*, 1999; Ikeguchi *et al*, 2000). However, these factors have not yet been sufficiently defined in patients with a high risk of cancer recurrence.

Several apoptosis inhibitors related to the baculovirus inhibitor of apoptosis (IAP) genes have recently been identified in human (Clem and Miller, 1994). Survivin belongs to a family of IAPs and has been shown to bind and inhibit the cell-death terminal effectors caspases-3 and -7, which induce apoptosis in cells (Mahotka *et al*, 1999). Strong survivin expression has been reported in several foetal tissues, whereas in adult tissues survivin transcripts have been detected only in the thymus and placenta. However, in the most common human cancers, survivin expression is again turned on (Adida *et al*, 1998). Recently, the clinical importance of *survivin* messenger RNA (mRNA) expression has been reported in various cancers including OSCC (Monzó *et al*, 1999; Sarela *et al*, 2000; Kato *et al*, 2001). However, these reports were based on the traditional reverse transcriptase polymerase chain reaction (RT–PCR) method. In this method, the real quantitative expression level of *survivin* mRNA cannot be estimated. To investigate whether *survivin* mRNA expression is a good indicator of cancer recurrence in OSCC, we used real-time RT–PCR method that enabled the real quantitative expression levels of *survivin* mRNA in each OSCCs to be evaluated.

## MATERIALS AND METHODS

### Cell line

The human OSCC cell line EC-GI-10 was purchased from Riken Gene Bank (Tsukuba Science City, Japan). EC-GI-10 was maintained in Dulbecco's modified Eagle's medium (GIBCO BRL, Grand Island, NY, USA) containing 10% foetal calf serum (GIBCO BRL) and 1% penicillin/streptomycin (GIBCO BRL) in a humidified atmosphere containing 5% CO_2_ at 37°C.

### Tissues

We obtained tumours and adjacent non-cancerous oesophageal epithelium samples from 57 patients with OSCC who underwent oesophagectomy between 1993 and 2000. Informed consent was obtained from all patients for subsequent use of their resected tissues. The present study conformed to the ethical standards of the World Medical Association Declaration of Helsinki. Tissue samples of approximately 0.1 g were collected immediately after resection of specimen. Non-cancerous tissues were obtained from regions distant from the tumours. Half of each tissue sample was fixed in 10% buffered formalin and embedded in paraffin. Sections (4 μm thick) were prepared for hematoxylin-eosin staining for histopathologic diagnosis and for immunohistochemical staining. The other half of the tissue was stored at −80°C until needed.

### Patients

The subjects (57 OSCC patients) included 54 men and three women, age at time of surgery was 65.2±1.2 years (mean±standard error; median, 67; range 45–84). Tumours were staged according to the TNM system. The stages of the 57 patients were Stage I (*n*=8), II (*n*=18), III (*n*=26), and IV (*n*=5). None of the patients received preoperative chemotherapy or radiation therapy. Transthoracic oesophagectomy was performed on 36 patients by right-sided antero-lateral thoracotomy and laparotomy. Intrathoracic and perigastric lymph nodes were dissected during this procedure. Transhiatal oesophagectomy without thoracotomy was performed on 19 patients. Lower oesophagectomy through the transabdominal approach was performed on two patients. Curative oesophagectomy was performed on 45 patients and non-curative oesophagectomy was performed on 12 patients (one: liver metastasis, three: extended lymph node metastasis, and eight: local invasion). All patients were followed until February 2002. The mean follow-up period for the 57 patients was 29.2 months (ranging from 1–99 months). Causes of death were determined from clinical findings. Nineteen patients were alive at February of 2002, and a total of 38 patients had died. Six patients died from diseases other than OSCC (four died from operative complications and the in-hospital mortality rate was 7%), while 32 died from recurrence or relapse of OSCC.

### Detection of* survivin* transcripts using real-time RT–PCR

Before starting the study, histopathologic examination confirmed that there were a sufficient number of cancer cells in the tumour samples and that no cancer cells had contaminated the non-cancerous tissues. Total RNA from EC-GI-10 and tissues was isolated using RNeasy Mini Kits (Qiagen, Hilden, Germany) according to the manufacturer's protocol. Total RNA was eluted with 50 μl of diethylpyrocarbonate (DEPC) water. RNA concentrations were determined by spectrophotometry. One microgram of the total RNA from each sample was heated to 60°C in a water bath for 10 min then cooled on ice for 2 min. Complementary DNA (cDNA) was synthesised with 1 μg of total RNA and 0.5 μg oligo (dT)_15_ primer (Promega, Madison, WI, USA) with Ready-to-Go^™^ You-Prime First-Strand Beads (Amersham Pharmacia Biotech Inc., Piscataway, NJ, USA). The beads contained moloney murine leukaemia virus reverse transcriptase (M-MuLV RT), 50 mM Tris-HCl (pH 8.3), 75 mM KCl, 7.5 mM DTT, 10 mM MgCl_2_, and 2.4 mM of each dNTP. Total volume was adjusted to 50 μl with DEPC water. The beads with the reaction mixture were incubated at 37°C for 60 min.

Primers and the TaqMan probes for *survivin* and *glyceraldehyde-3-phosphate dehydrogenase* (*GAPDH*) (*survivin*: forward primer; 5′-TGCCCCGACGTTGCC-3′, reverse primer; 5′-CAGTTCTTGAATGTAGAGATGCGGT-3′, and probe; 5′-CCTGGCAGCCCTTTCTCAAGGACC-3′, *GAPDH*: forward primer; 5′-GAAGGTGAAGGTCGGAGTC-3′, reverse primer; 5′-GAAGATGGTGATGGGATTTC-3′, and probe; 5′-CAAGCTTCCCGTTCTCAGCC-3′) were synthesised according to a previous report (Wen *et al*, 2000). Specific oligonucleotide probes were labelled with a reporter fluorescent dye (FAM (6-carboxy-fluorescein)) at the 5′ end and a quencher fluorescent dye (TAMRA (6-carboxy-tetramethyl-rhodamine)) at the 3′ end. AmpliTaq DNA polymerase extended the primer and displaced the TaqMan probe through its 5′–3′ exonuclease activity. No signal was emitted when the probe was intact.

### Quantification of gene expression

**Figure 1:**
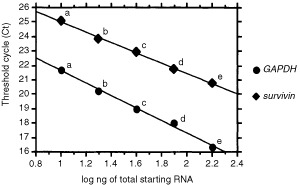
Standard curves for *survivin* and *GAPDH*. The plots represent the log of the input amount (log ng of total starting RNA of EC-GI-10; a: 160 ng, b: 80 ng, c: 40 ng, d: 20 ng, e: 10 ng) as the *x*-axis and threshold cycle (Ct) as the *y*-axis for *survivin* and *GAPDH*.

Quantification of gene expression was performed by real-time quantitative RT–PCR (Gene Amp® 5700 Sequence Detection System (Perkin-Elmer Applied Biosystems, Foster City, CA, USA)), which uses the 5′ nuclease activity of Taq polymerase to detect PCR amplicons (Yajima *et al*, 1998; Mitas *et al*, 2001). The PCR solution (50 μl) was composed of 1 μl of cDNA solution, 5 pmol of the forward and reverse primers, 10 pmol of internal probe, and TaqMan Universal PCR Master Mix. PCR was carried out after incubation at 50°C for 2 min, denaturing at 95°C for 10 min, 45 cycles of 95°C for 15 s, and 61°C for 1 min. Experiments were performed in duplicate for each data point. For each reaction tube, the fluorescence signal of the reporter dye (FAM) was divided by the fluorescence signal of the passive reference dye (TAMRA) to obtain a ratio defined as the normalised reporter signal (Rn). The threshold line was set at an Rn of 0.05 (10 standard deviations above the mean of the baseline fluorescence emission calculated from cycles 3–20) (Yajima *et al*, 1998). The point at which the amplification plot crossed this threshold was defined as Ct, which represented the cycle number at this point. Standard curves for *survivin*, and *GAPDH* were generated using serial dilution (containing 160, 80, 40, 20 and 10 ng) of total RNA derived from the EC-GI-10 cell line. The plots represent the log of the input amount (log ng of total starting RNA) as the *x*-axis and Ct as the *y*-axis. Equations were derived from the lines of the calibration curves (Yajima *et al*, 1998). The formulas for *survivin* and *GAPDH* were as follows: *survivin*, *y*=28.6–3.6*x* (*r*^2^=0.998) and *GAPDH*, *y*=26.3–4.5*x* (*r*^2^=0.991) (Figure 1

). For each of the experimental samples, the amount of *survivin* and *GAPDH* mRNAs were determined from the standard curves. *GAPDH* transcripts were monitored as a control to quantify the transcripts of the genes in each sample. The normalised amounts of *survivin* mRNA, respectively, were determined by dividing the amount of *survivin* mRNA by the amount of *GAPDH* mRNA for each sample.

### Immunohistochemistry

To identify survivin protein expressing cells, tissue samples (tumours and non-cancerous oesophageal epitheliums) were immunostained with a rabbit anti-human survivin polyclonal antibody (diluted to 1 : 20; Alpha Diagnostic International, San Antonio, TX, USA). After overnight incubation the slides with a primary antibody at 4°C, ENVISION+ (DAKO Japan Co. Ltd., Kyoto, Japan) was applied as the secondary antibody for 30 min. The reaction products were visualised with diaminobenzidine as the chromogen, and the slides were counterstained with methyl green.

### Statistical analysis

The χ^2^ and Fishers exact probability tests were used to compare the distribution of individual variables among the patient groups. Differences in the numerical data between the two groups were evaluated using the Mann-Whitney U test. Differences in the numerical data among more than three groups were evaluated using the Kruskal–Wallis test. Survival rates were calculated using the Kaplan–Meier method. The log rank test was used for comparisons of two survival curves. The influence of each variable on mode of recurrence was assessed by the multivariate logistic regression analysis. A *P*-value of 0.05 was considered to be statistically significant.

## RESULTS

### *Survivin/GAPDH* ratio in tumours and in non-cancerous tissue of the oesophagus

**Figure 2:**
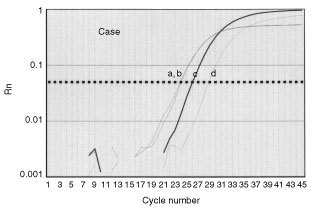
Amplification curves of *survivin* and *GAPDH* in a typical case. Line a: *GAPDH* of tumour, line b: *GAPDH* of non-cancerous tissue adjacent to carcinoma, line c: *survivin* of tumour, and line d: *survivin* of non-cancerous tissue. The horizontal line at Rn=0.05 is the threshold for detection.

The *survivin*/*GAPDH* ratio of carcinomas (mean±standard error (s.e.): 5.3±0.8, range: 0.4–37) was significantly higher than that of non-cancerous tissues (3.6±0.8, range: 0–37.5, *P*=0.0003). Figure 2

shows one typical case. In this case, the *survivin*/*GAPDH* ratio of the tumour tissue was 3-fold higher than that of non-cancerous tissue. A significant positive correlation between tumour-*survivin*/*GAPDH* ratios and non-cancerous tissue-*survivin*/*GAPDH* ratios was detected in 57 patients (ρ=0.413, *P*=0.0022). However, the *survivin*/*GAPDH* ratio of tumours did not correlate with histologic type, lymph node involvement, or depth of tumour invasion (Table 1

**Table 1:**
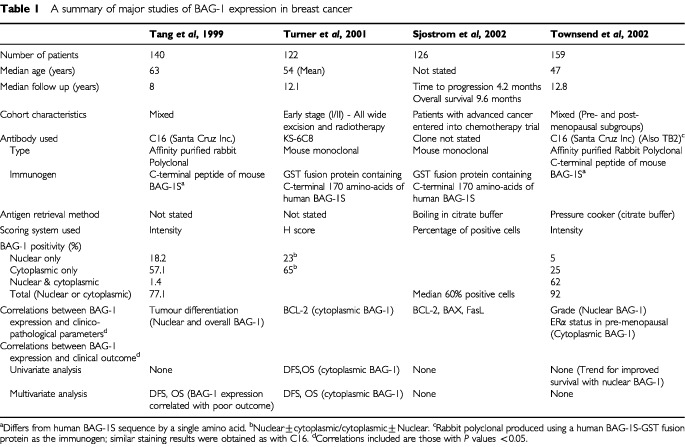
Clinicopathological findings of patients and tumor-*survivin/GAPDH* mRNA ratios

).

### Localisation of survivin protein in oesophageal tissue

**Figure 3:**
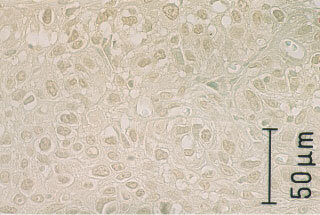
Strong survivin protein expression was detected in nuclei of all cancer cells and weak survivin expression was observed in the cytoplasm by immunostaining with a polyclonal antibody (×100).

**Figure 4:**
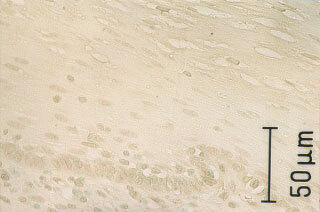
Normal esophageal cells in the basal layer show weak survivin protein expression (×100).

Survivin protein was detected not only in the cytoplasm, but also in nuclei of all cancer cells (Figure 3

) and normal basal cells in non-cancerous epithelium. Normal basal cells showed weak survivin protein expression (Figure 4

). Sixteen tumours expressed strong survivin protein, while the remaining 41 tumours expressed weak survivin protein as same level as normal basal cells. The *survivin*/*GAPDH* ratio of 16 tumours with strong survivin protein expression (11.5±2.1) was significantly higher than that of 41 tumours with weak expression (2.9±0.3, *P*<0.0001).

### Prognostic significance of the* survivin/GAPDH* ratio in OSCC

**Figure 5:**
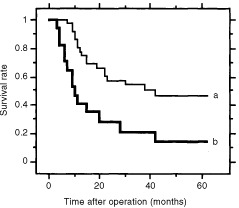
The disease-specific survival curves of the 53 surviving patients. The survival curve of 17 with high-*survivin*/*GAPDH* ratio-tumours (line b) was significantly lower than that of 36 with low-*survivin*/*GAPDH* ratio- tumors (line a). The difference between two survival curves was statistically significant (*P*=0.0018).

We divided the 57 patients into two subgroups according to their tumour-*survivin*/*GAPDH* ratio. The cut-off value of the *survivin*/*GAPDH* ratio of tumours was decided from the formula (mean+2×s.e.) of 57 non-cancerous tissue-*survivin*/*GAPDH* ratios (Cheung and Cheung, 2001). Mean *survivin*/*GAPDH* ratio of 57 non-cancerous tissues was 3.6 and s.e. was 0.8. Thus, the calculated cut-off value of *survivin*/*GAPDH* ratio of tumours was 5.2. Eighteen tumours showed high *survivin*/*GAPDH* ratio (>5.2) and 13 of these 18 (72%) showed strong survivin protein expression by immunohistochemistry. While in 39 tumours with low *survivin*/*GAPDH* ratio (⩽5.2), only three tumours (7.7%) showed strong protein expression. Figure 5

shows the survival curves of the 53 surviving patients. The survival curve of 17 patients with high-*survivin*/*GAPDH* ratio-tumours (>5.2, 5-year survival rate was 14.1%) was significantly lower than that of 36 patients with low-*survivin*/*GAPDH* ratio-tumours (⩽5.2, 5-year survival rate was 46.8%, *P*=0.0018). In February 2002, in the 53 surviving patients, tumour recurrence or relapse was detected in 33 (62.3%) and 32 had died from OSCC. The mean tumour-*survivin*/*GAPDH* ratio of the 33 who had developed recurrent or relapsed tumours (6.3±1.2) was higher than that of the 20 without tumour recurrence or relapse (3.6±0.7, *P*=0.0281). The clinicopathological and the biological factors, thought to be correlated with the occurrence of cancer recurrence, were analysed. By multivariate logistic regression analysis, the tumour-*survivin*/*GAPDH* ratio was detected as a risk factor of cancer recurrence independent from depth of tumour invasion or lymph node involvement (Table 2

**Table 2:**
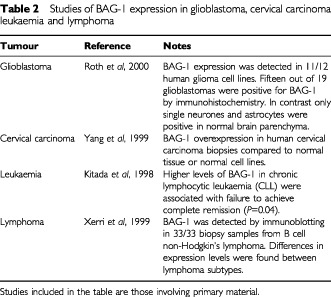
Factors affecting tumour recurrence analysed by multivariate logistic regression analysis

).

In the 53 surviving patients, adjuvant radiation therapy was performed for 36 patients (total dose was 30 to 50 Grays). The remaining 17 patients were excluded from adjuvant radiation therapy because of high age, cardio-pulmonary complication, or early stage of tumour. The 5-year survival rate of the 36 patients who underwent adjuvant radiation therapy (32.8%) was not different from that of the 17 patients who did not (45.8%, *P*=0.3317). The 5-year survival rates of patients with high-tumour-*survivin*/*GAPDH* ratio (with adjuvant therapy, *n*=8, 0%; without adjuvant therapy, *n*=9, 22.2%) were significantly poorer than those of patients with low-tumour-*survivin*/*GAPDH* ratio (with adjuvant therapy, *n*=28, 40.1%, *P*=0.0014; without adjuvant therapy, *n*=8, 72.9%, *P*=0.0253).

## DISCUSSION

The role of survivin protein is unique, having been shown to bind specifically to caspases-3 and -7 and to inhibit apoptosis *in vitro* system (Tamm *et al*, 1998). Furthermore, Li *et al* (1998) reported that survivin expresses during the G2/M phase of the cell cycle and the disruption of survivin-microtubule interactions results in increased caspase-3 activity and accelerated apoptotic cell death. Ito *et al* (2000) reported that hepatocellular carcinoma cell lines transfected with survivin show a significant decrease in cells in the G0/G1 phase and an increase in cells in the S and G2M phases. These findings indicate that survivin protein expression may correlate not only with reduced apoptotic cell death but also with an increased proliferative activity of cancer cells. Recently, the clinical importance of *survivin* mRNA expression has been reported in various cancers. These reports demonstrated that the prognosis of patients with *survivin* mRNA expression positive tumours was significantly worse than that of patients with *survivin* negative.

The reports noted above were based on the RT–PCR method, while in the present study we used real-time RT–PCR to evaluate the quantitative expression levels of *survivin* mRNA in oesophageal tissues instead of either the RT–PCR or Northern blot methods. The expression levels of *GAPDH* (internal control) mRNA were reported to be quite different among samples, even when the same amounts of total RNAs were used (Suzuki *et al*, 2001). This phenomenon might be overlooked in Northern blot or RT–PCR assay. Moreover, Northern blot requires many complicated techniques such as gel electrophoresis, membrane transfer, and hybridisation and requires a comparatively long time to obtain results. Also, it is difficult to estimate the real amount of gene expression level by a RT–PCR method. The recent development of real-time RT–PCR technology has made reliable and accurate PCR quantification possible. This technology monitors the entire PCR reaction by fluorescence detection, thereby allowing the beginning of the exponential phase of amplification (threshold cycle) to be measured. This reaction point is considered the most reliable point of the PCR reaction relative to sample concentration (Wittwer *et al*, 1997).

Using real-time RT–PCR method, we found that the mean *survivin* mRNA expression level of non-cancerous oesophageal tissues (the normalised amounts of *survivin* mRNA were determined by dividing the amounts of mRNA levels by the amount of *GAPDH* mRNA for each sample) was 3.6. Kato *et al* (2001) reported that *survivin* mRNA expression of normal oesophageal tissues detected by RT–PCR method was observed in 47.1% of cases and that this rate was higher than that of normal lung tissues (12%) or that of normal colorectal tissues (29.1%). It is well known that esophageal epithelium has rapid renewal (Takubo *et al*, 1997). These findings indicate that *survivin* mRNA may express with high proliferative activity even in normal tissues.

Our results demonstrated that tumour-*survivin* mRNA expression level did not correlate with lymph node metastasis or depth of tumour invasion in OSCC. The same result has been observed in colorectal carcinoma (Sarela *et al*, 2000) and in non-small-cell lung cancer (Monzó *et al*, 1999). These findings indicate that tumour-*survivin* mRNA expression level may not correlate with metastatic potential of tumour cells.

However, in previous studies, and in ours, patients who had tumours with high *survivin* mRNA expression revealed shorter survival. Recently, it has been shown that pancreatic cancer cells or gastric cancer cells with strong *survivin* mRNA expression showed a chemo-radioresistance *in vitro* (Asanuma *et al*, 2000; Ikeguchi *et al*, 2002). Moreover, from a clinical perspective, patients with *survivin* mRNA positive tumours showed chemo-radioresistance in OSCC (Kato *et al*, 2001). In a study of neuroblastoma (Azuhata *et al*, 2001), and in our study of OSCC, tumour-*survivin* mRNA expression showed strong positive correlations with the tumour recurrence of patients. From these results, it is suggested that micrometastatic foci with strong *survivin* mRNA expression may have high proliferative activity and may increase rapidly and also may acquire resistance to adjuvant chemo-radiotherapy.

We conclude that *survivin* mRNA expression may become a useful prognostic biomarker in OSCC and the quantitative real-time RT–PCR method is a useful technique for determining gene expression levels in tissue.
